# Urinary Biomarkers of Brain Diseases

**DOI:** 10.1016/j.gpb.2015.08.005

**Published:** 2016-01-02

**Authors:** Manxia An, Youhe Gao

**Affiliations:** 1 Department of Pathophysiology, State Key Laboratory of Medical Molecular Biology, Institute of Basic Medical Sciences, Chinese Academy of Medical Sciences, Beijing 100005, China; 2 School of Basic Medicine, Peking Union Medical College, Beijing 100005, China; 3 Department of Biochemistry and Molecular Biology, Beijing Normal University, Beijing Key Laboratory of Gene Engineering and Biotechnology, Beijing 100875, China

**Keywords:** Brain diseases, Urine, Proteomics, Metabolomics, Biomarkers

## Abstract

**Biomarkers** are the measurable changes associated with a physiological or pathophysiological process. Unlike blood, **urine** is not subject to homeostatic mechanisms. Therefore, greater fluctuations could occur in **urine** than in blood, better reflecting the changes in human body. The roadmap of **urine** biomarker era was proposed. Although **urine** analysis has been attempted for clinical diagnosis, and **urine** has been monitored during the progression of many diseases, particularly urinary system diseases, whether **urine** can reflect brain disease status remains uncertain. As some **biomarkers** of **brain diseases** can be detected in the body fluids such as cerebrospinal fluid and blood, there is a possibility that **urine** also contain **biomarkers** of **brain diseases**. This review summarizes the clues of **brain diseases** reflected in the **urine** proteome and metabolome.

## Introduction

The use of effective biomarkers has great significance for the prediction, diagnosis, monitoring, treatment, and prognosis of many diseases. Urine is not subject to homeostatic mechanisms and accommodate many changes that may reflect status of the body, such as pregnancy, aging, and daily rhythms [1]. These changes may be used as promising biomarkers [2]. Therefore the roadmap of urine indicator as the future of biomarker applications was proposed [3]. Currently, most studies on urinary biomarkers have focused on kidney diseases due to the close relationship between the kidneys and urine [4], [5]. The lack of attention to urinary biomarkers in other diseases, like brain diseases, may be due to the fact that anatomically, the brain and urine are not closely related and there exists the filtering effect of the blood–brain barrier and the kidneys. Most brain disease studies have focused on cerebrospinal fluid (CSF) and blood [6], [7], in which changes caused by brain diseases may be attenuated by homeostatic mechanisms. However, the potential presence of brain disease biomarkers in urine is largely neglected, and there are only a few urinary biomarker studies on brain diseases. Although the brain and urine are distantly apart and a direct relationship is hard to establish with current scientific knowledge, we cannot rule out the possibility that changes present in the brain are somehow reflected in urine. Here, we summarize urinary proteomics and metabolomics applications for the study on brain diseases, which may be useful for researchers interested in exploring the field.

Omics technology including proteomics and metabolomics has garnered a lot of attention recently in the biomarker field thanks to its high-throughput and high-sensitivity capacities. Urine has been commonly examined in omics fields. Proteins in the urine mainly come from blood by filtration and the kidneys/urinary tracts by secretion. It was previously believed that urine contains very few proteins. However, a recent study indicated that the content of urinary proteins from normal subjects is 0–0.8 g/l [8]. Nonetheless, urine contains fewer high abundance proteins than blood, which makes the identification of urinary proteins relatively easy since no high abundance suppression exists. Six thousand proteins can be identified in urine, indicating the complexity of the urine proteome and highlighting the availability of the urine proteome for the study of biomarkers (personal communication). Currently, urinary proteomics is mainly used to search for biomarkers of kidney diseases, such as IgA nephropathy [9], renal cell carcinoma [10], and kidney transplant [11]. However, urinary proteomics can also be used to study a variety of diseases, such as sleep apnea [12], eclampsia [13], and cardiovascular diseases [14].

Through urinary metabolomics, small molecule metabolites in urine with molecular weights less than 1000 Da can be comprehensively analyzed. Metabolites in urine may reflect state of the body to some extent and serve as informative biomarkers for some diseases. Urinary metabolomics has been widely used in the studies of various diseases, including diabetic nephropathy [15], acute kidney injury [16], chronic heart failure [17], liver cancer [18], and breast cancer [19].

The main purpose of the biomarker studies is to identify specific and stable biomarkers for a particular disease. Because many diseases are heterogeneous, finding a single biomarker relative to one disease is difficult. Thus, a panel of proteins or metabolites identified by omics technology is considered feasible and would be desirable biomarkers of the disease. In addition, the search for brain disease biomarkers in urine may pose challenges because their changes in the urine are sensitive and can be affected by many factors transiently. However, only stable changes that are strongly associated with disease can be effective biomarkers. Such changes can only be considered specific if they are independent of impact of transient factors. In the current review, we summarized previous studies searching brain diseases biomarkers in urine by proteomics and metabolomics.

## Neuropsychiatric disorders

### Major depressive disorder

Major depressive disorder (MDD) is a severe neuropsychiatric disorder that is characterized by disordered mood, cognition, neurovegetative functions, and mental activity [20]. Clinically, the diagnosis of MDD is mainly based on a subjective symptom assessment, while no objective and effective measurements are available so far [21]. Therefore, biomarkers that can enhance traditional symptom-based assessments and predict treatment response are greatly needed in clinical settings.

In order to search biomarkers of MDD, urine of 42 first-episode drug-naïve MDD subjects and 28 normal subjects was analyzed using a peptidomics method, which mainly targeted the small polypeptides. Five peptides (*m*/*z* 8307.22, 3222.17, 4640.35, 5072.14, and 1196.47) were selected from the 29 putative urinary peptides for establishing a candidate classification model. This model showed good diagnostic performance for MDD with a 90.5% sensitivity (38/42), a 92.9% specificity (26/28), and a 91.4% accuracy (64/70). Four of these five peptides have been identified to correspond to four known proteins, including serum albumin (*m*/*z* 1196.47), alpha-1-microglobulin/bikunin precursor (AMBP, *m*/*z* 3222.17), heparan sulfate proteoglycan (HSPG, *m*/*z* 4640.35), and apolipoprotein A-I (APOA1) (*m*/*z* 5072.14) [22]. Among them, altered expression of APOA1 has previously been correlated with several other psychiatric disorders [23], [24].

In addition, urinary metabolomics was also used for MDD biomarker identification. Urinary metabolomes of a training set (82 first-episode, drug-naïve MDD subjects and 82 normal subjects) were measured using nuclear magnetic resonance (NMR) [25]. Metabolites associated with tricarboxylic acid (TCA) cycle, intestinal microflora metabolism, and tryptophan-nicotinic acid pathway were altered in the MDD patients’ urine. A receiver-operating characteristic (ROC) curve analysis was performed to evaluate the validity of these possible biomarkers. A biomarker classification model consisting of formate, malonate, *N*-methylnicotinamide, m-hydroxyphenylacetate, and alanine showed the highest predictive power for MDD, in which the area under the ROC curve (AUC) of this classification model was 0.81 in the training samples and 0.89 in the test samples. This specific classification model also performed well in a test set despite of a lack of age matching. In a follow-up study, urine from the same MDD and control subjects were analyzed by gas chromatography-mass spectrometry (GC–MS) [26]. Six metabolites (sorbitol, uric acid, azelaic acid, hippuric acid, quinolinic acid, and tyrosine) were selected from 23 differential metabolites and considered promising biomarkers for MDD diagnosis (0.905 of AUC in training samples and 0.837 of AUC in test samples). A composite model add *N*-methylnicotinamide, which was identified in a previous study [25], to the six aforementioned metabolites performed even better (AUC: 0.909 in training samples and 0.917 in test samples) compared to the current model with six metabolites only in distinguishing MDD patients from controls [25]. Metabolic pathway analysis showed disturbances in peripheral glucose metabolism, perturbed kynurenine pathway metabolism, disturbances in the tyrosine-phenylalanine pathway, and increased oxidative stress in MDD patients. These two studies identified some candidate urinary biomarkers with the potential for diagnosing MDD in the future clinical setting and demonstrated the power of the metabolomics-based method for searching MDD biomarkers. However, large samples from a heterogeneous population are needed to validate the applicability and sensitivity of these candidate diagnostic models. The correlation between these differential metabolites and MDD must also be further investigated to understand the pathological mechanisms and therapeutic targets.

### Bipolar disorder

Bipolar disorder (BD) is a debilitating and life-long psychiatric disorder, affecting up to 1% of the population worldwide [27]. The pathogenesis of BD remains unclear [28], and currently, BD is mainly diagnosed according to the subjective identification of disease symptoms.

The aforementioned metabolites azelaic acid and *N*-methylnicotinamide in urine are also candidate biomarkers for BD. In order to searching biomarkers of BD, the urinary metabolomes of 78 BD patients and 43 normal subjects were analyzed by combining NMR and GC–MS [29]. A biomarker panel containing five metabolites (azelaic acid, 2,4-dihydroxypyrimidine, β-alanine, pseudouridine, and α-hydroxybutyrate) was established as a candidate diagnostic model. The AUC of this model was 0.974 in the training set and 0.964 in the test set, which suggest a solid performance. This biomarker model had better accuracy than previous ones with GC–MS or NMR metabolomics separately, suggesting that the integration of these two technologies can be used to obtain a more comprehensive and reliable biomarker model of BD than using either method alone [30], [31]. Most of the patients recruited in these studies underwent treatment with anti-psychotic medications. Nonetheless, the influence of medications on metabolome profiles has not been evaluated, which however would presumably make it more difficult to find specific biomarkers for BD.

It is of note that BD patients exhibit gender-specific phenotypes, such as the differences in the age of onset and lobular volumes [32], [33]. Chen et al. thus examined gender-specific BD urinary biomarkers using NMR-based metabolomics [34]. A male-specific biomarker model (α-hydroxybutyrate, choline, formate, and *N*-methylnicotinamide) and a female-specific biomarker model (α-hydroxybutyrate, oxalacetate, acetone, and *N*-methylnicotinamide) were differentially established to distinguish BD subjects of different genders from their healthy counterparts. It is probably not surprising that the male-specific biomarker model poorly distinguished female patients from controls, and the female-specific biomarker model poorly distinguished male patients from controls. Other than the metabolites mentioned above, several other metabolites are present at different levels in urine between the male and female BD patients. Therefore, gender-specific studies may identify more sensitive biomarkers for accurate diagnosis.

### Autistic spectrum disorders

Autistic spectrum disorders (ASDs) represent a complex series of neurodevelopmental diseases and are usually diagnosed in the first three years of life [35]. Because there are no objective tests for diagnosis, clinical signs are commonly used, such as social skill deficits, communication deficits, and deficiency in play skills.

Yap et al. compared urinary metabolomes of ASD children and healthy controls using NMR method [36]. The alterations in mammalian-microbial cometabolites as well as metabolites associated with nicotinic acid metabolism were mainly observed in ASD children. In addition, the levels of several amino acids in urine, including taurine, glutamate, and *N*-acetyl glycoprotein fragments were altered in ASD children as well. Additionally, perturbations in gut microbiota, including *Clostridium* sp., have been revealed in ASD children in previous study [37]. Furthermore, increased concentrations of several organic acids and sugars, such as 3-(3-hydroxyphenyl)-3-hydroxypropanoic acid, five-carbon sugars, and ribose, were detected in the urine of ASD children in a GC–MS-based metabolomics study, whereas concentrations of fructose, 1,2,3-butanetriol, and propylene glycol were markedly decreased in the urine of ASD children relative to controls [38]. Meanwhile, increased concentrations of 3-(3-hydroxyphenyl)-3-hydroxypropanoic in urine have been found in children diagnosed with ASD or schizophrenia [39], which may derive from m-tyrosine, a bacterial metabolite that can lead to symptoms of autism in rats [40]. Perturbation of organic acid and sugar levels in urine of ASD children was also found in another study [41], indicating that these metabolites have the potential to serve as biomarkers of ASD and may help in ASD diagnosis, identification of subtypes, and search for potential therapeutic targets.

### Schizophrenia

Schizophrenia is a severe emotional disorder characterized by a retreat from reality with the formation of delusions [42]. There are no effective objective diagnostic methods for this disease yet. Cai et al. analyzed the global metabolomic profile and the specific neurotransmitter metabolites in the urine of schizophrenia subjects (first-episode neuroleptic-naïve) and normal subjects. Urine samples were taken before and after 6-week of risperidone monotherapy [43]. The concentrations of several neurotransmitter metabolites, such as glucosamine, glutamic acid, and vanilmandelic acid, were altered in the urine of patients. In addition, the concentrations of creatinine, α-KG, citrate, valine, and glycine were altered in urine in schizophrenia patients as well. These findings suggest abnormalities in energy and amino acid metabolism in schizophrenia patients. In another study, serum and urine samples from schizophrenic subjects and normal subjects were analyzed by a combination of NMR and MS [44]. A compound biomarker model using five serum metabolites (glycerate, eicosenoic acid, β-hydroxybutyrate, pyruvate, and cystine) and one urinary metabolite (β-hydroxybutyrate) was identified. This model can distinguish schizophrenic subjects from normal subjects with a good accuracy (AUC = 1). In addition, levels of fatty acids and ketone bodies in the serum and urine were increased, indicating that glucose deficiency in the brain of schizophrenic patients may possibly result in increased fatty acid catabolism.

These studies established the foundation of exploiting the laboratory-based diagnostic tests for psychiatric disorders and have identified some candidate urinary biomarkers (Table 1). However, the sample sizes (no more than two hundred) recruited in these studies were relatively small. Therefore, subsequent larger-scale clinical studies are needed before applying to clinical settings.

## Cerebrovascular disorders

### Stroke

Stroke is a common degenerative disease with high mortality and morbidity. It is characterized by a variety of neurological symptoms, resulting from sudden blockage or bleeding of brain blood vessels [45]. Currently, the clinical assessment of stroke is based on clinical signs supplemented by imaging such as computed tomography (CT) and magnetic resonance imaging (MRI). The accuracy of CT is approximately 82% at 6 h of cerebral ischemia, which unfortunately is beyond the therapeutic window for intravenous recombinant tissue plasminogen activator (tPA) [46]. On the other hand, the false negative rate of MRI is as high as 17% and cannot be applied to patients with pacemaker, metal stents, and other implants [47].

The first study on the urinary proteomic biomarkers for stroke was performed by using 2-DE to examine the impact of salt loading on the protein abundance in both urine and serum of three rat strains: Wistar-Kyoto rats, spontaneously hypertensive stroke-prone rats, and spontaneously hypertensive rats [48]. It was found in SHRSP rats that several proteins, including hemopexin, albumin, transferrin, α_2_-HS-glycoprotein, kallikrein-binding protein, α_1_-antitrypsin, Gc-globulin, and transthyretin, appeared in urine prior to the clinically apparent stroke symptom after a period of salt loading. These data suggest possible early stroke diagnosis based on urinary proteomics. In a later study, urinary proteomes of stroke patients and healthy controls were analyzed by using capillary electrophoresis (CE)-MS. As a result, two biomarker models each consisting of 14 biomarkers (AUC 0.75) or 35 biomarkers (AUC 0.86) were established [49]. These biomarkers showed good distinguishing ability between patients and normal controls in the test samples. Furthermore, a classifier based on 34 potential biomarkers selected from differential molecules was strongly correlated with the severity of stroke. However, the reliability of this study may be compromised by the presence of several confounding factors that possibly affect renal function in patients and controls, including diabetes mellitus, hypertension, and the use of some medications, such as alpha-blocker, calcium channel blocker. Nonetheless, this was the first attempt to search urinary biomarkers of stroke using MS-based proteomics, providing significant clues for future studies.

Urinary metabolomics can also be employed to search for stroke biomarkers. For instance, Jung et al. found that the urine metabolome of stroke patients differed from that of healthy controls [50]. The major alterations in urinary metabolites of stroke subjects include reduced concentrations of dimethylamine, glycine, and hippurate. Decrease in these metabolites was associated with folic acid deficiency and hyperhomocysteinemia, which have been reported to be associated with an increased risk of both cognitive impairment and stroke [51]. These studies identified some candidate biomarkers (Table 2) for diagnosis and provided some insights into the possible pathogenesis of stroke. More technology platforms may also be applied in future to investigate the comprehensive proteic and metabolic characteristics of stroke and search for effective biomarkers.

## Neurodegenerative diseases

Neurodegenerative diseases include Alzheimer’s disease (AD), Parkinson’s disease (PD), multiple sclerosis (MScl), and transmissible spongiform encephalopathies (TSEs). Early intervention and treatment can effectively slow down the progress of these diseases. Therefore, there is an urgent need to find effective biomarkers for the prediction, diagnosis, monitoring, and treatment of these diseases.

### Alzheimer’s disease

AD is a chronic degenerative disease, characterized by extracellular amyloid plaques resulting from the accumulation of amyloid β proteins and intracellular neurofibrillary tangle due to the aggregation of tau proteins [52]. Diagnosis of AD is based on subjective neuropsychological tests supplemented by late-stage biomarkers in CSF [53]. The early diagnosis may be important before the irreversible brain damage or mental decline has occurred. Therefore there is a serious need for a convenient and accurate method for early diagnosis of AD.

Two studies reported the screening of urinary metabolic biomarkers of AD using different animal models and technologies. Fukuhara et al. analyzed the urinary metabolome of Tg2576 transgenic AD mice using NMR [54]. 3-Hydroxykynurenine, homogentisate, and tyrosine were identified as effective biomarkers of AD prior to dementia onset, whereas 1-methylnicotinamide, 2-oxoglutarate, citrate, urea, dimethylamine, trigonelline, and trimethylamine were identified as effective biomarkers of late-stage AD. The elevated levels of proteins associated with oxidative stress, including 3-hydroxykynurenine, homogentisate, and allantoin, in 4-month-old AD mice indicated that oxidative stress occurs before the appearance of obvious symptoms of AD. Later on, another study performed the urinary metabolomic biomarker screening using liquid chromatography–MS (LC–MS) with isotope labeling in the TgCRND8 transgenic AD mice in comparison to normal mice [55]. An obvious difference in the urinary metabolite composition was observed between TgCRND8 transgenic AD mice and normal mice at 15–17 weeks of age. Such change became even more apparent after 25 weeks and was associated with phenotypic changes in the AD mice. As a result, methionine, 5-hydroxyindoleacetic acid, desaminotyrosine, taurine, and N1-acetylspermidine were identified as promising biomarkers. Taurine is the only biomarker molecule commonly identified in the two aforementioned studies, suggesting that different biomarkers could be identified with varied methods even for the same disease.

### Parkinson’s disease

PD is a debilitating neurological disorder of unknown origin and is mainly characterized by movement difficulties [56]. The neuropathological hallmarks of PD include the notable loss and denaturation of dopaminergic neurons in the substantia nigra, as well as the appearance of Lewy bodies [56]. Currently, the pathological mechanisms of PD remain unknown and there are no effective early biomarkers for PD diagnosis.

Urinary metabolites of 106 idiopathic PD patients were compared with those of 104 normal control subjects using the metabolomics method combined with high-performance liquid chromatography (HPLC) and LC–MS [57]. The PD patients exhibited distinct urinary metabolomic characteristics significantly different from controls. There are significant differences between PD patients and healthy controls in the levels of several metabolites that are associated with fatty acid beta-oxidation, phenylalanine metabolism, histidine metabolism, steroidogenesis, tryptophan metabolism, nucleotide metabolism, and tyrosine metabolism, such as hydroxylauroylcarnitine, phenylacetic acid, histidine, dihydrocortisol, and acetylserotonin. In addition, abnormality in the kynurenine pathway in tyrosine metabolism has been detected in the alpha-synuclein overexpressing fly model of PD, indicating that the kynurenine pathway may be associated with the pathological mechanism of PD.

### Multiple sclerosis

MScl is an inflammatory demyelinating autoimmune diseases characterized by paralysis as well as impairment of language, motor, and visual ability [58]. Symptoms of MScl are often relieved during the 3rd trimester of pregnancy and aggravated during the first postpartum period [59]. Urinary proteins from these two periods of pregnancy in MScl patients were analyzed by using LC–MS [60]. Changes in concentrations of 531 peptides from MScl subjects and 36 peptides from normal subjects were detected during the 3rd trimester relative to those in the postpartum period. Among them, 43 peptides which were altered in MScl patients in relative to controls, were considered disease-related. The concentrations of several immunomodulatory function proteins, such as placenta-derived pregnancy-associated immunoregulatory proteins and choriogonadotropin subunit beta variant 1 (CGB1), were significantly elevated in the urine of patients during the 3rd trimester of pregnancy comparing with the postpartum period and the controls. Elevated levels of these pregnancy-related proteins may reflect the decrease in severity of MScl during the 3rd trimester of pregnancy, indicating that alterations in urinary proteins in MScl patients may reflect changes in the remote central nervous system (CNS) and even pathological changes in the brain.

### Transmissible spongiform encephalopathies

TSEs are a group of genetic, infectious, or sporadic degenerative nervous system disorders associated with abnormal prions that can be propagated in different species and the aggregation of prion protein (PrPd) in the CNS is important for the evaluation of TSEs [61]. However, the PrPd quantification is difficult to apply because brain tissue is hard to obtain.

Urinary proteomics can aid in the diagnosis of TSEs as shown in two studies. Promising biomarkers found in these two studies are summarized in Table 2. In one of the studies, urinary proteomes of bovine spongiform encephalopathy (BSE)-infected cattle and healthy cattle were compared by using 2-DE [62]. Although confounding factors, such as breed, gender, and age, could influence the protein composition of urine, disease-specific alterations in urinary proteins identified by 2-DE can still be distinguished in clinical-stage infected cattle from normal cattle with good accuracy. In the other study, Lamoureux et al. further verified the usability of urine to test for TSEs. They analyzed the urinary proteome of scrapie-infected mice, in comparison with that of AD mice and controls [63] and found differences in the concentrations of some urinary proteins could be used for TSE tests and could even distinguish TSEs from AD. These disease-specific proteins were primarily related to the formation of desmosomes and neurodegenerative disease [64], [65], [66]. These studies about neurodegenerative diseases have found some promising biomarkers by using samples from patients or animal models (Table 2).

## Neuroendocrine neoplasm and traumatic brain injury

Neuroendocrine neoplasm (NEN) is a group of heterogeneous tumors that originate from peptidergic neurons and neuroendocrine cells [67]. NEN can occur in many organs and tissues in the body. The majority of NEN are formed in the small bowel and pancreas. One study analyzed the urinary metabolites of 28 NEN patients and 17 healthy controls by using NMR analysis [68]. Alterations in urinary metabolomes could be used to distinguish NEN patients from controls with good accuracy and these differential metabolites performed better in the monitoring of metastases of NEN compared with CgA alone (AUC 0.86 *vs.* 0.80), which is a common marker used to monitor the metastasis of tumors. In future studies, metabolites related to the severity of NEN should be analyzed to find representative metabolites of different disease stages. The fine management of patients can then be conducted according to the severity of patients.

Traumatic brain injury (TBI) is one of the common fatal and disabling diseases in the clinical setting [69]. One study analyzed the urinary signature of five TBI patients upon admission to acute brain injury rehabilitation and five normal control subjects [70]. As a result, 2476 discriminant variables were reproducibly identified. Although the numbers of samples involved in this study was too small to exclude variations among individuals, this study indicates that detecting urinary signatures may serve as an alternative approach in the diagnosis and monitoring of rehabilitative TBI.

These two studies preliminarily demonstrate the possibility of searching biomarker of brain tumors and injury in urine. These promising biomarkers are summarized in Table 3.

## Concluding remarks and perspectives

Urine, as a promising source of disease biomarkers, was largely ignored in brain disease field compared with other body fluids. Nonetheless, the limited studies on urinary-based biomarker of brain diseases provide evidence that urine may be a good source for searching biomarkers of some brain diseases. These studies show that the levels of some discriminating molecules in the urine are different between brain disease groups and control groups. They should be verified by subsequent large-scale clinical samples before potential application to clinical settings, since there are only a few validation studies with large clinical samples at this moment.

There are some confounding factors in the aforementioned studies, which would compromise the reliability of the results. These include the medications, diet, and the lifestyle of patients. As one of the factors that may influence urinary signatures, medication should be taken into account [71]. Two studies concerning MDD conducted by Xie’s group eliminated the influence of medication by using samples from first-episode, drug-naïve MDD patients in the training set [25], [26]. BMI, age, and gender were also strictly controlled between the MDD group and the healthy control group in the training set of these studies, which increased the reliability of the results. In addition, components in human urine also can be affected by other unrelated factors, such as diet and life style [72], [73]; these factors are difficult to balance due to the limited sample size. Using large samples size or animal models might tackle this problem to some extent. Through the use of animal models, changes influenced by single pathological factors can be detected; such studies can provide more closely related biomarkers associated with specific pathological changes of disease. A small number of model animals can be analyzed efficiently to provide preliminary potential biomarkers, which can then be verified in clinical samples [3].

The same candidate biomarkers may be applied to closely related diseases. For instance, increased levels of azelaic acid and reduced levels of *N*-methylnicotinamide concentration were detected in the urine of both MDD and BD patients compared with controls [25], [26], [29], [31], [34]. Additionally, the concentration of uric acid in urine increased in both MDD and schizophrenia patients comparing to controls [26], [43]. Decreased citrate levels in urine were found in NEN, stroke, and schizophrenia patients compared with controls [43], [50], [68]. These common biomarkers may reflect shared pathological processes in these diseases. Disease-specific biomarkers should be distinguished from general biomarkers of neurological diseases to provide convenient and efficient methods for diagnosis, prognosis, and treatment of respective diseases. Most studies that search for brain disease biomarkers in urine compared patients with one particular disease to healthy controls, instead of patients with similar diseases. The urinary signatures of multiple similar diseases should be compared in a single study to identify similar pathophysiologies and find specific biomarkers.

The studies reviewed in this article provided some promising evidences, indicating that clinically applicable urine biomarkers of brain diseases may largely exist and should be useful in future diagnoses. Larger clinical studies also are required to verify these clues. Urine biomarkers of brain diseases would be used and more convenient for clinical application if proved to be reliable. Inferred by these existing studies mentioned above, searching brain disease biomarkers in urine would open new way to find more brain disease biomarkers.

## Competing interests

The authors have declared that there are no competing interests.

## Figures and Tables

**Table 1:** Summary of urinary biomarkers of human neuropsychiatric disorders

**Disease**	**Method**	**No. of patients**	**No. of controls**	**Potential biomarkers**		**Ref.**
**Upregulated**	**Downregulated**
MDD	MALDI-TOF MS-based peptidomics	66	41	Serum albumin, apolipoprotein A-I, HSPG	Protein AMBP	[22]
NMR-based metabolomics	134	126	Formate, alanine	Malonate, *N*-methylnicotinamide, m-hydroxyphenylacetate	[25]
GC–MS-based metabolomics	134	126	Sorbitol, uric acid, azelaic acid	Hippuric acid, quinolinic acid, tyrosine	[26]

BD	NMR and GC–MS-based metabolomics	71	126	Azelaic acid, β-alanine, α-hydroxybutyrate	Pseudouridine, 2,4-dihydroxypyrimidine	[29]
GC–MS-based metabolomics	71	94		2,4-Dihydroxypyrimidine	[30]
NMR-based metabolomics	86	96	α-Hydroxybutyrate, isobutyrate	Choline, *N*-methylnicotinamide	[31]
NMR-based metabolomics	42 male	53 male	α-Hydroxybutyrate, formate	Choline, *N*-methylnicotinamide	[34]
NMR-based metabolomics	44 female	43 female	α-Hydroxybutyrate	Oxalacetate, acetone, *N*-methylnicotinamide	[34]

ASD	NMR-based metabolomics	39	62	*N*-methyl-2-pyridone-5-carboxamide, *N*-methyl nicotinic acid, taurine, *N*-methyl nicotinamide	Glutamate	[36]
NMR-based metabolomics	21	21	3-(3-Hydroxyphenyl)-3-hydroxypropanoic, 3,4-dihydroxybutyric acid, glycolic acid, glycine, *cis*-aconitic acid	Fructose, 1,2,3-butanetriol, propylene glycol	[38]
GC–MS-based metabolomics	14	10	Oxalic acid, β-hydroxybutyric acid, ribonic acid, m-hydroxybenzoic acid	Phosphoric acid, sebacic acid	[41]

Schizophrenia	MS/MS, UPLC–MS and NMR-based metabolomics	11	11	Uric acid, pregnanediol, valine, glycine, glucose	Creatinine, hippurate, creatine, citrate, α-KG, taurine, TMAO	[43]
GC-TOF and NMR-based metabolomics	112	120	β-Hydroxybutyrate		[44]

*Note:* MDD, major depressive disorder; BD, bipolar disorder; ASD, autism spectrum disorder; HSPG, basement membrane-specific heparan sulfate proteoglycan core protein; AMBP, alpha-1-microglobulin/bikunin precursor; α-KG, α-ketoglutaric acid; TMAO, trimethylamine-*N*-oxide.

**Table 2:** Summary of urinary biomarkers of neurodegenerative diseases

**Disease**	**Method**	**Species**	**No. of subjects**	**No. of controls**	**Potential biomarkers**		**Ref.**
**Upregulated**	**Downregulated**
Stroke	2-DE-based proteomics	Rat	8 stroke-prone spontaneously-hypertensive rats	5 Wistar-Kyoto rats 5 spontaneously hypertensive rats	Transferrin, hemopexin, albumin, transthyretin, α_2_-HS-glycoprotein, Gc-globulin, α_1_-antitrypsin, kallikrein-binding protein		[48]
CE-MS-based proteomics	Human	101	48	Classifiers contains 14 biomarkers or 35 biomarkers		[49]
NMR-based metabolomics	Human	28	30		Citrate, dimethylamine, creatinine, glycine, hippurate	[50]

AD	NMR-based metabolomics	Mouse	3–5 each for 4, 10, 15 months	3–5	3-Hydroxykynurenine, homogentisate, allantoin	Dimethylamine, trimethylamine	[54]
LC–MS-based metabolomics	Mouse	12	12	Methionine, desaminotyrosine	*N*^1^-acetylspermidine, 5-hydroxyindoleacetic acid	[55]

PD	LC–MS-based metabolomics	Human	106	104	Cortisol,11-deoxycortisol, 21-deoxycortisol, histidine, urocanic acid, imidazoleacetic acid, hydroxyphenylacetic acid		[57]

MScl	LC–MS-based proteomics	Human	31	8	Levels of 531 peptides from MScl patients and 36 peptides from controls were altered in the third trimester compared to the postpartum period, 43 peptides are associated with MScl		[60]

TSEs	2-DE-based proteomics	Cattle	13	14	9 spots in 2-DE		[62]
2-DE and LC–MS-based proteomics	Mouse	4 scrapie mice	4 controls and 4 Alzheimer mice at each time point	Classifier contains 20 spots		[63]

*Note:* AD, Alzheimer’s disease; PD, Parkinson’s disease; MScl, multiple sclerosis; TSEs, transmissible spongiform encephalopathies.

**Table 3:** Summary of urinary biomarkers of human brain tumors and injury

**Disease**	**Method**	**No. of patients**	**No. of controls**	**Potential biomarkers**		**Ref.**
**Upregulated**	**Downregulated**
NEN	NMR-based metabolomics	28	17		Citrate, hippurate	[68]

TBI	MS-based metabolomics	5	5	2476 discriminant variables		[70]

*Note:* NEN, neuroendocrine neoplasms; TBI, traumatic brain injury.

## References

[1] Wu J., Gao Y. (2015). Physiological conditions can be reflected in human urine proteome and metabolome. Expert Rev Proteomics.

[2] Gao Y. (2013). Urine-an untapped goldmine for biomarker discovery?. Sci China Life Sci.

[3] Gao Y. (2014). Roadmap to the urine biomarker era. MOJ Proteomics Bioinform.

[4] Schanstra J.P., Mischak H. (2015). Proteomic urinary biomarker approach in renal disease: from discovery to implementation. Pediatr Nephrol.

[5] Pejcic M., Stojnev S., Stefanovic V. (2010). Urinary proteomics–a tool for biomarker discovery. Ren Fail.

[6] Jove M., Portero-Otin M., Naudi A., Ferrer I., Pamplona R. (2014). Metabolomics of human brain aging and age-related neurodegenerative diseases. J Neuropathol Exp Neurol.

[7] Miao Y., Liao J.K. (2014). Potential serum biomarkers in the pathophysiological processes of stroke. Expert Rev Neurother.

[8] Lerma E.V. (2008). Approach to the patient with renal disease. Prim Care.

[9] Haubitz M., Wittke S., Weissinger E.M., Walden M., Rupprecht H.D., Floege J. (2005). Urine protein patterns can serve as diagnostic tools in patients with IgA nephropathy. Kidney Int.

[10] Wu D.L., Zhang W.H., Wang W.J., Jing S.B., Xu Y.M. (2008). Proteomic evaluation of urine from renal cell carcinoma using SELDI-TOF-MS and tree analysis pattern. Technol Cancer Res Treat.

[11] Quintana L.F., Sole-Gonzalez A., Kalko S.G., Banon-Maneus E., Sole M., Diekmann F. (2009). Urine proteomics to detect biomarkers for chronic allograft dysfunction. J Am Soc Nephrol.

[12] Seetho I.W., Siwy J., Albalat A., Mullen W., Mischak H., Parker R.J. (2014). Urinary proteomics in obstructive sleep apnoea and obesity. Eur J Clin Invest.

[13] Carty D.M., Siwy J., Brennand J.E., Zürbig P., Mullen W., Franke J. (2011). Urinary proteomics for prediction of preeclampsia. Hypertension.

[14] Delles C., Diez J., Dominiczak A.F. (2011). Urinary proteomics in cardiovascular disease: achievements, limits and hopes. Proteomics Clin Appl.

[15] Ng D., Salim A., Liu Y., Zou L., Xu F., Huang S. (2012). A metabolomic study of low estimated GFR in non-proteinuric type 2 diabetes mellitus. Diabetologia.

[16] Xie G., Zheng X., Qi X., Cao Y., Chi Y., Su M. (2010). Metabonomic evaluation of melamine-induced acute renal toxicity in rats. J Proteome Res.

[17] Kang S.M., Park J.C., Shin M.J., Lee H., Oh J., Ryu do H. (2011). (1)H nuclear magnetic resonance based metabolic urinary profiling of patients with ischemic heart failure. Clin Biochem.

[18] Shariff M.I., Ladep N.G., Cox I.J., Williams H.R., Okeke E., Malu A. (2010). Characterization of urinary biomarkers of hepatocellular carcinoma using magnetic resonance spectroscopy in a Nigerian population. J Proteome Res.

[19] Slupsky C.M., Steed H., Wells T.H., Dabbs K., Schepansky A., Capstick V. (2010). Urine metabolite analysis offers potential early diagnosis of ovarian and breast cancers. Clin Cancer Res.

[20] Hill R.J., Chopra P., Richardi T. (2012). Rethinking the psychogenic model of complex regional pain syndrome: somatoform disorders and complex regional pain syndrome. Anesth Pain Med.

[21] Fava M., Kendler K.S. (2000). Major depressive disorder. Neuron.

[22] Wang Y., Chen J., Chen L., Zheng P., Xu H.B., Lu J. (2014). Urinary peptidomics identifies potential biomarkers for major depressive disorder. Psychiatry Res.

[23] Puchades M., Hansson S.F., Nilsson C.L., Andreasen N., Blennow K., Davidsson P. (2003). Proteomic studies of potential cerebrospinal fluid protein markers for Alzheimer’s disease. Brain Res Mol Brain Res.

[24] Huang J.T., Wang L., Prabakaran S., Wengenroth M., Lockstone H.E., Koethe D. (2008). Independent protein-profiling studies show a decrease in apolipoprotein A1 levels in schizophrenia CSF, brain and peripheral tissues. Mol Psychiatry.

[25] Zheng P., Wang Y., Chen L., Yang D., Meng H., Zhou D. (2013). Identification and validation of urinary metabolite biomarkers for major depressive disorder. Mol Cell Proteomics.

[26] Zheng P., Chen J.J., Huang T., Wang M.J., Wang Y., Dong M.X. (2013). A novel urinary metabolite signature for diagnosing major depressive disorder. J Proteome Res.

[27] Merikangas K.R., Jin R., He J.P., Kessler R.C., Lee S., Sampson N.A. (2011). Prevalence and correlates of bipolar spectrum disorder in the world mental health survey initiative. Arch Gen Psychiatry.

[28] Muller-Oerlinghausen B., Berghofer A., Bauer M. (2002). Bipolar disorder. Lancet.

[29] Chen J.J., Liu Z., Fan S.H., Yang D.Y., Zheng P., Shao W.H. (2014). Combined application of NMR- and GC–MS-based metabonomics yields a superior urinary biomarker panel for bipolar disorder. Sci Rep.

[30] Xu X.J., Zheng P., Ren G.P., Liu M.L., Mu J., Guo J. (2014). 2,4-Dihydroxypyrimidine is a potential urinary metabolite biomarker for diagnosing bipolar disorder. Mol BioSyst.

[31] Zheng P., Wei Y.-D., Yao G.-E., Ren G.-P., Guo J., Zhou C.-J. (2013). Novel urinary biomarkers for diagnosing bipolar disorder. Metabolomics.

[32] Grigoroiu-Serbanescu M., Nothen M.M., Ohlraun S., Propping P., Maier W., Wickramaratne P. (2005). Family history influences age of onset in bipolar I disorder in females but not in males. Am J Med Genet B Neuropsychiatr Genet.

[33] Mackay C.E., Roddick E., Barrick T.R., Lloyd A.J., Roberts N., Crow T.J. (2010). Sex dependence of brain size and shape in bipolar disorder: an exploratory study. Bipolar Disord.

[34] Chen J.J., Huang H., Zhao L.B., Zhou D.Z., Yang Y.T., Zheng P. (2014). Sex-specific urinary biomarkers for diagnosing bipolar disorder. PLoS One.

[35] Muhle R., Trentacoste S.V., Rapin I. (2004). The genetics of autism. Pediatrics.

[36] Yap I.K., Angley M., Veselkov K.A., Holmes E., Lindon J.C., Nicholson J.K. (2010). Urinary metabolic phenotyping differentiates children with autism from their unaffected siblings and age-matched controls. J Proteome Res.

[37] Parracho H.M., Bingham M.O., Gibson G.R., McCartney A.L. (2005). Differences between the gut microflora of children with autistic spectrum disorders and that of healthy children. J Med Microbiol.

[38] Noto A., Fanos V., Barberini L., Grapov D., Fattuoni C., Zaffanello M. (2014). The urinary metabolomics profile of an Italian autistic children population and their unaffected siblings. J Matern Fetal Neonatal Med.

[39] Shaw W. (2010). Increased urinary excretion of a 3-(3-hydroxyphenyl)-3-hydroxypropionic acid (HPHPA), an abnormal phenylalanine metabolite of Clostridia spp. in the gastrointestinal tract, in urine samples from patients with autism and schizophrenia. Nutr Neurosci.

[40] Dyck L.E., Kazakoff C.W., Dourish C.T. (1982). The role of catecholamines, 5-hydroxytryptamine and m-tyramine in the behavioural effects of m-tyrosine in the rat. Eur J Pharmacol.

[41] Kaluzna-Czaplinska J., Zurawicz E., Struck W., Markuszewski M. (2014). Identification of organic acids as potential biomarkers in the urine of autistic children using gas chromatography/mass spectrometry. J Chromatogr B Analyt Technol Biomed Life Sci.

[42] Lieberman J.A. (1999). Is schizophrenia a neurodegenerative disorder? A clinical and neurobiological perspective. Biol Psychiatry.

[43] Cai H.L., Li H.D., Yan X.Z., Sun B., Zhang Q., Yan M. (2012). Metabolomic analysis of biochemical changes in the plasma and urine of first-episode neuroleptic-naive schizophrenia patients after treatment with risperidone. J Proteome Res.

[44] Yang J., Chen T., Sun L., Zhao Z., Qi X., Zhou K. (2013). Potential metabolite markers of schizophrenia. Mol Psychiatry.

[45] Donnan G.A., Fisher M., Macleod M., Davis S.M. (2008). Stroke. Lancet.

[46] Latchaw R.E., Alberts M.J., Lev M.H., Connors J.J., Harbaugh R.E., Higashida R.T. (2009). Recommendations for imaging of acute ischemic stroke: a scientific statement from the American Heart Association. Stroke.

[47] Chalela J.A., Kidwell C.S., Nentwich L.M., Luby M., Butman J.A., Demchuk A.M. (2007). Magnetic resonance imaging and computed tomography in emergency assessment of patients with suspected acute stroke: a prospective comparison. Lancet.

[48] Sironi L., Tremoli E., Miller I., Guerrini U., Calvio A.M., Eberini I. (2001). Acute-phase proteins before cerebral ischemia in stroke-prone rats: identification by proteomics. Stroke.

[49] Dawson J., Walters M., Delles C., Mischak H., Mullen W. (2012). Urinary proteomics to support diagnosis of stroke. PLoS One.

[50] Jung J.Y., Lee H.S., Kang D.G., Kim N.S., Cha M.H., Bang O.S. (2011). 1H-NMR-based metabolomics study of cerebral infarction. Stroke.

[51] Homocysteine Studies C (2002). Homocysteine and risk of ischemic heart disease and stroke: a meta-analysis. JAMA.

[52] Mucke L. (2009). Neuroscience: Alzheimer’s disease. Nature.

[53] Lovestone S. (2010). Searching for biomarkers in neurodegeneration. Nat Med.

[54] Fukuhara K., Ohno A., Ota Y., Senoo Y., Maekawa K., Okuda H. (2013). NMR-based metabolomics of urine in a mouse model of Alzheimer’s disease: identification of oxidative stress biomarkers. J Clin Biochem Nutr.

[55] Peng J., Guo K., Xia J., Zhou J., Yang J., Westaway D. (2014). Development of isotope labeling liquid chromatography mass spectrometry for mouse urine metabolomics: quantitative metabolomic study of transgenic mice related to Alzheimer’s disease. J Proteome Res.

[56] Lang A.E., Lozano A.M. (1998). Parkinson’s disease. First of two parts. N Engl J Med.

[57] Luan H., Liu L.F., Meng N., Tang Z., Chua K.K., Chen L.L. (2015). LC–MS-based urinary metabolite signatures in idiopathic Parkinson’s disease. J Proteome Res.

[58] Compston A., Coles A. (2002). Multiple sclerosis. Lancet.

[59] Confavreux C., Hutchinson M., Hours M.M., Cortinovis-Tourniaire P., Moreau T. (1998). Rate of pregnancy-related relapse in multiple sclerosis. Pregnancy in Multiple Sclerosis Group. N Engl J Med.

[60] Singh V., Stingl C., Stoop M.P., Zeneyedpour L., Neuteboom R.F., Smitt P.S. (2015). Proteomics urine analysis of pregnant women suffering from multiple sclerosis. J Proteome Res.

[61] Prusiner S.B. (1991). Molecular biology of prion diseases. Science.

[62] Plews M., Lamoureux L., Simon S.L., Graham C., Ruddat V., Czub S. (2011). Factors affecting the accuracy of urine-based biomarkers of BSE. Proteome Sci.

[63] Lamoureux L., Simon S.L., Plews M., Ruddat V., Brunet S., Graham C. (2013). Urine proteins identified by two-dimensional differential gel electrophoresis facilitate the differential diagnoses of scrapie. PLoS One.

[64] Ferone G., Mollo M.R., Thomason H.A., Antonini D., Zhou H., Ambrosio R. (2013). P63 control of desmosome gene expression and adhesion is compromised in AEC syndrome. Hum Mol Genet.

[65] Li X., Masliah E., Reixach N., Buxbaum J.N. (2011). Neuronal production of transthyretin in human and murine Alzheimer’s disease: is it protective?. J Neurosci.

[66] Kuhr F., Lowry J., Zhang Y., Brovkovych V., Skidgel R.A. (2010). Differential regulation of inducible and endothelial nitric oxide synthase by kinin B1 and B2 receptors. Neuropeptides.

[67] Massironi S., Sciola V., Peracchi M., Ciafardini C., Spampatti M.P., Conte D. (2008). Neuroendocrine tumors of the gastro-entero-pancreatic system. World J Gastroenterol.

[68] Kinross J.M., Drymousis P., Jimenez B., Frilling A. (2013). Metabonomic profiling: a novel approach in neuroendocrine neoplasias. Surgery.

[69] Ghajar J. (2000). Traumatic brain injury. Lancet.

[70] Ottens A.K., Stafflinger J.E., Griffin H.E., Kunz R.D., Cifu D.X., Niemeier J.P. (2014). Post-acute brain injury urinary signature: a new resource for molecular diagnostics. J Neurotrauma.

[71] Li X., Zhao M., Li M., Jia L., Gao Y. (2014). Effects of three commonly-used diuretics on the urinary proteome. Genomics Proteomics Bioinformatics.

[72] Andersen M.B., Kristensen M., Manach C., Pujos-Guillot E., Poulsen S.K., Larsen T.M. (2014). Discovery and validation of urinary exposure markers for different plant foods by untargeted metabolomics. Anal Bioanal Chem.

[73] Airoldi L., Magagnotti C., Iannuzzi A.R., Marelli C., Bagnati R., Pastorelli R. (2009). Effects of cigarette smoking on the human urinary proteome. Biochem Biophys Res Commun.

